# CircPTP4A2 (hsa_circ_0007364) facilitates non-small cell lung cancer progression by regulating miR-127-5p/SMC3

**DOI:** 10.1186/s13062-026-00815-2

**Published:** 2026-05-07

**Authors:** Yali Feng, Jiang Hong, Changgang Yang, Chun Cheng, Yujie Xue, Jiaqi Zhang, Yu Lu, Xiang Cao, Gengxi Jiang, Xiaodan Chong

**Affiliations:** 1 Department of Oncology, Haian People’s Hospital, Nantong, China; 2 Department of Thoracic Surgery, Naval Medical University Affiliated Changhai Hospital, Shanghai, China; 3 Department of Cardiothoracic Surgery, Hai’an People’s Hospital, Nantong, China; 4 Department of Thoracic Surgery, Tumor Hospital Affiliated to Nantong University, Nantong, China; 5 Department of Pathology, The Affiliated Huaian NO. 1 People’s Hospital of Nanjing Medical University, Huaian, Jiangsu China; 6 Department of Pathology, Taizhou People’s Hospital Affiliated to Dalian Medical University, Taizhou, China; 7 The Second People’s Hospital of Nantong, Nantong, China; 8 Department of Thoracic Surgery, Nantong First People’s Hospital, Nantong, China; 9 Department of Cardiothoracic Surgery, Affiliated Hospital and Medical School of Nantong University, Nantong, China; 10 Clinical Cancer Institute, Translational Medicine Center, Naval Medical University, Shanghai, 200433 China

**Keywords:** NSCLC, circRNA, circPTP4A2, miR-127-5p, SMC3

## Abstract

Non-small cell lung cancer (NSCLC) is a major subtype of lung cancer and accounts for a large proportion of cancer-related deaths worldwide. Despite extensive research progress in recent years, the diagnosis and treatment of lung cancer remain insufficient. There is an urgent need to deepen the mechanistic understanding of lung cancer, develop early diagnostic strategies, and explore novel therapeutic targets. In this study, qRT-PCR was used to detect the expression of circPTP4A2 (circular RNA PTP4A2) in tumor and adjacent normal tissues from 50 NSCLC patients. CircPTP4A2 was significantly upregulated in tumor tissues and was closely associated with patient survival and prognosis. In vitro silencing of circPTP4A2 in NSCLC cell lines SPCA1 and H1299 significantly inhibited cell proliferation and malignant metastatic potential. Moreover, modulating the expression of miR-127-5p and SMC3 effectively reversed the phenotypic changes induced by circPTP4A2 knockdown. In conclusion, circPTP4A2 is upregulated in NSCLC and promotes tumorigenesis and progression through the miR-127-5p/SMC3 signaling axis.

**Supplementary Information:**

The online version contains supplementary material available at 10.1186/s13062-026-00815-2.

## Background

Lung cancer is one of the most common malignant tumors worldwide and poses a serious threat to human health. It remains the leading cause of cancer-related death globally [1–3]. Based on pathological classification, lung cancer is divided into two main types: small-cell lung cancer (SCLC) and non-small-cell lung cancer (NSCLC) [4–6]. Despite continuous improvements in lung cancer diagnosis and therapy, more than 80% of NSCLC cases exhibit high metastatic potential and drug resistance, leading to poor clinical outcomes [7]. Therefore, although considerable progress has been made in recent research, the diagnosis and treatment of lung cancer are still inadequate. A better scientific understanding, reliable early diagnosis, and novel therapeutic targets are urgently needed.

Circular RNAs (circRNAs), a class of non-coding RNAs, are characterized by a covalent bond linking the 3’and 5′ends generated by back-splicing [8, 9]. Most circRNAs are generated through non-canonical alternative splicing mediated by spliceosomes or group I/II ribozymes [10]. Genome-wide RNA sequencing analyses have revealed that circRNAs are evolutionarily conserved and abundantly expressed [11–13]. CircRNAs can be classified as exonic, intronic, or exon–intron circRNAs according to their genomic origin. Functionally, many circRNAs act as endogenous miRNA sponges by harboring miRNA-binding sites to sequester and inhibit miRNA activity [14]. Besides, circRNAs also function as RNA-binding protein (RBP) sequestering agents as well as transcription regulators to modulate gene expressions [15]. Increasing evidence indicates that circRNAs are closely associated with human diseases, especially cancers, and serve as promising biomarkers due to their high abundance and stability [16–20].

A previous study reported that circPTP4A2 (hsa_circ_0007364) promotes cervical cancer progression via the miR-101-5p/MAT2A axis [21]. However, its biological function and underlying mechanism in NSCLC remain unclear. CircInteractome prediction suggested that circPTP4A2 could directly target and sponge miR-127-5p. miR-127-5p is downregulated in various cancers and acts as a tumor suppressor. miRDB further predicted that miR-127-5p targets the structural maintenance of chromosomes 3 (SMC3) gene [22, 23]. SMC3 belongs to the SMC protein family and is a core component of the cohesin complex, which mediates sister chromatid cohesion during mitosis to ensure accurate chromosome segregation [24–27]. The SMC family (SMC1–6) maintains chromosomal stability by forming heterodimers and is widely reported to play critical roles in tumor development. SMC3 is highly expressed in lung cancer, and targeting SMC3 can inhibit lung cancer progression [28, 29]. In pancreatic cancer, epigenetic silencing of SMC3 regulates Rab27a expression and drives tumor progression [30].

In this study, we demonstrated that silencing circPTP4A2 in SPCA1 and H1299 cells significantly inhibited cell proliferation and metastasis in vitro. Intervention with miR-127-5p or SMC3 effectively rescued the phenotypic changes caused by circPTP4A2 knockdown. Collectively, our results confirm that circPTP4A2 is upregulated in NSCLC and promotes tumor development and progression through the miR-127-5p/SMC3 regulatory axis.

## Materials and methods

### Sample collection

Totally, 50 patients with NSCLC admitted at the Changhai Hospital Affiliated to Naval Medical University. All patients were newly diagnosed with NSCLC without previous history of malignancies and did not receive any anticancer therapies before admission. Patients diagnosed with multiple clinical conditions were excluded. This study was approved by the Ethics Committee of Changhai Hospital Affiliated to Naval Medical University (Approval No.: CHEC2021-061) and conducted in accordance with the Declaration of Helsinki. NSCLC and paired adjacent non-tumor tissues were collected by lung biopsy and verified by histopathological examination. All samples were immediately stored at − 80 °C. Written informed consent was obtained from all participants.

### Target gene prediction

The CircInteractome database (https://circinteractome.nia.nih.gov/) was used to predict miRNAs targeted by circPTP4A2. The miRDB database (http://mirdb.org/) was used to predict mRNAs targeted by miR-127-5p.

### qPCR and reverse transcription polymerase chain reaction (RT-PCR)

Total RNA was isolated using Trizol Reagent (Takara). For cDNA synthesis, 1 µg of total RNA was reverse-transcribed with the PrimeScript™ RT reagent Kit (Takara) according to the manufacturer’s protocols. Quantitative PCR (qPCR) was performed using the TB Green Premix Ex Taq kit (TaKaRa) on a StepOne Plus Real-Time PCR System (Applied Biosystems). 18sRNA and GAPDH served as internal controls. Relative RNA expression levels were analyzed using the 2(-△△CT) method. Primers used in this study are listed in Table S1.

### RNase R treatment

A total of 5 µg total RNA was incubated with 10 units of RNase R (Epicentre Technologies) at 37 °C for 15 min. Following treatment, RNA samples underwent RT-PCR to evaluate the expression levels of target RNAs.

### Subcellular localization

Cytoplasmic and nuclear RNAs were isolated using the NE-PER Reagent (Thermo Scientific) following the manufacturer’s protocols. The expression level of circPTP4A2 in each fraction was then quantified by qRT-PCR, with U6 and GAPDH serving as control markers for nuclear and cytoplasmic RNA, respectively.

### Cell culture and transfection

SPCA1 and H1299 cell lines were cultured in DMEM medium containing 10% fetal bovine serum (FBS, Gibco) and 100 mg/mL streptomycin (Gibco). Transfection procedures were performed using the DharmaFECT1 transfection reagent (Dharmacon) following the manufacturer’s protocols. All cellular experiments were independently replicated three times. Transfection efficiency was evaluated by qRT-PCR 24 h after transfection to confirm the knockdown or overexpression efficiency of the corresponding targets.

### Colony formation assay

SPCA1 and H1299 cells were transfected and then seeded at a density of 500 cells per well in 6-well plates, followed by incubation at 37 °C in a 5% CO₂ atmosphere. After 14 days, cell colonies were fixed and stained with 0.1% crystal violet solution. Following two washes with PBS (phosphate-buffered solution, Invitrogen), the number of cell colonies was manually counted.

### Transwell assay

To assess cell migration rates under varying treatment conditions, a transwell assay with Matrigel was performed. Transwell chambers (Corning, New York, USA) were first coated overnight with 200 mg/mL Matrigel (BD Biosciences, SanJose, USA). Approximately 5 × 10⁵ transfected SPCA1 or H1299 cells were then suspended in serum-free RPMI1640 medium. The upper chamber was loaded with 200 µL of the cell suspension, while the lower chamber received 500 µL of RPMI1640 medium supplemented with 10% FBS. After 24 h of incubation, non-invaded cells on the upper surface of the chamber were removed using a cotton swab. Migrated cells attached to the underside of the transwell membrane were fixed with 95% ethanol and stained with 0.1% crystal violet buffer. Finally, the number of invaded cells was counted under a phase-contrast microscope (Olympus, Tokyo, Japan). For the migration assay (without Matrigel), the same protocol was followed except for omitting the Matrigel coating step on the upper chamber.

### Western blot

Proteins were extracted using RIPA lysis buffer (Thermo Scientific, MA, USA) containing protease and phosphatase inhibitor cocktail (Amresco, cat PB0425-5G). Protein quantification was performed with the Pierce™ BCA Protein Assay Kit (Thermo Scientific, cat 23227). Equal protein aliquots were then separated by SDS-PAGE and transferred to a PVDF membrane (Bio-Rad, CA, USA). After overnight incubation with primary antibodies at 4 °C, the membrane was washed three times with TBS-T buffer and incubated with HRP-conjugated anti-mouse or anti-rabbit IgG for 1 h at room temperature. Membrane signals were detected using an ImageQuant LAS 4000mini (Bio-Rad). Primary antibodies against BCL2 (Invitrogen, Cat. No. MA5-41210), BAX (Abcam, Cat. No. ab270742), cleaved-caspase 9 (Abcam, Cat. No. ab32042), and GAPDH (Abcam, Cat. No. ab9482), SMC3(Abcam, Cat. ab.AB9263).

### RNA immunoprecipitation (RIP) assay

RIP assay was performed using a Magna RIP RNA-Binding Protein Immunoprecipitation Kit (Merck Millipore, Germany) according to the instructions of the manufacturer. The quality and abundance of circPTP4A2 were detected by RT-qPCR.

### Biotinylated RNA pull-down assay

The protocol for RNA pull-down assay was adopted from William et al.25 with few modifications. Briefly, biotin-labeled circPTP4A2 probes and truncated oligo probes (control probes) were incubated with streptavidin magnetic beads for 1 h at room temperature to form probe-magnetic bead complex. Afterward, the SPCA1 and H1299 cell lysates were incubated with the probe-bead complex for 30 min for the binding of RNA-associated proteins to RNAs. The RNA-protein complexes on the beads were subsequently washed three times and eluted. QRT-PCR was then performed to assess the miR-127-5P relative expression level in the collected fractions.

### In vivo xenograft tumor models

Female BALB/c nude mice (10–18 g, 4 weeks old) were purchased from the Shanghai National Laboratory Animal Center (Shanghai, China). For the experiment, 1 × 10⁷ H1299 cells with stable expression of oligo or sh-circPTP4A2 were subcutaneously injected into the right flank of each 4-week-old female nude BALB/c mouse, with six mice per group using a random number table, and the grouping process was completed by an independent researcher who did not participate in the subsequent experimental operations and result detection. In the process of tumor volume measurement and result analysis, the researcher was blinded to the grouping information to avoid subjective bias. Tumor growth was monitored weekly, and tumor volume was calculated using the formula: volume (mm³) = (length × width²)/2. After 35 days, mice were humanely euthanized via cervical dislocation. All animal experiments were approved by the Ethics Committee of Changhai Hospital Affiliated to Naval Medical University and strictly followed the Guide for the Care and Use of Laboratory Animals of Changhai Hospital Affiliated to Naval Medical University.

### Statistical analysis

The GSE158695 dataset was downloaded from the Gene Expression Omnibus (GEO) database (https://www.ncbi.nlm.nih.gov/geo/) and the raw data (CEL files) of the GSE158695 dataset were processed using the R software (version 4.3.2). The limma R package was employed to analyze circRNA expression profiles in cancer and para-cancer tissues. The screening criteria were |log2(fold change)| > 1 and adjusted p-value < 0.05. Student’s t-test was used for comparisons between two groups. One-way ANOVA followed by Tukey’s post-hoc multiple-comparison test was used for comparisons among more than two groups. Pearson’s rank correlation test was used to calculate the correlation coefficient between gene expression levels, while the Kaplan–Meier (KM) method was applied to assess the association between patient survival rates and circPTP4A2 expression. A significance threshold of *p* < 0.05 was used to define statistically significant differences.

## Results

### Characterization and identification of circPTP4A2 in NSCLC

**Fig. 1:**
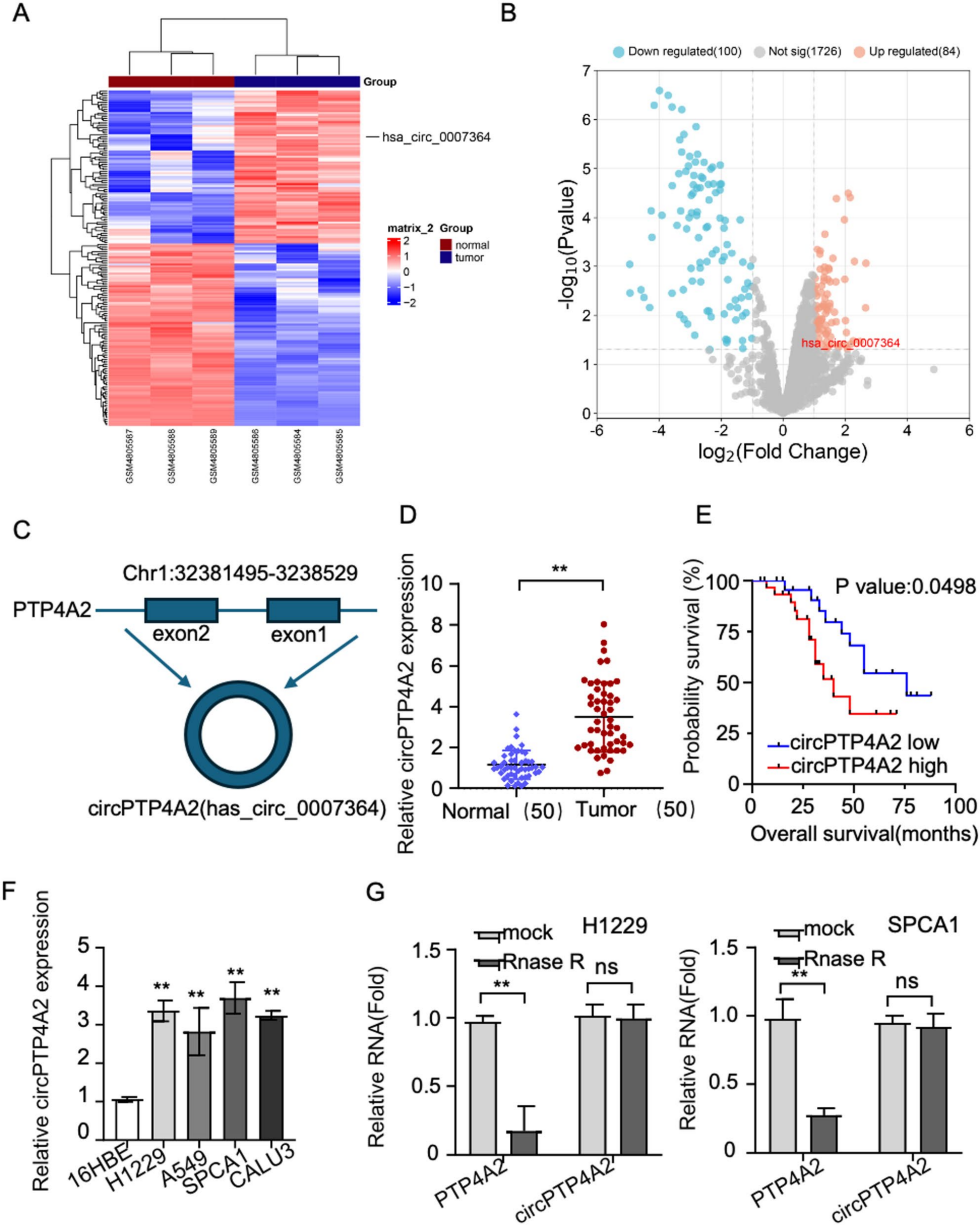
Characterization and identification of circPTP4A2 in NSCLC. **A-B**, Heatmap and volcano plot revealing upregulated and downregulated circRNAs in the GEO data (GSE158695), including circPTP4A2 (|log FC|≥1), *p* < 0.05). **C**, CircPTP4A2 formation diagram. **D**, CircPTP4A2 expression was detected by qRT-PCR in 50 pairs of NSCLC and normal tissues. **E**, Kaplan-Meier analysis of 50 patients with non-small cell lung cancer showing high circPTP4A2 expression predicted worse overall survival (*p* = 0.0498). **F**, circPTP4A2 expression in human bronchial epithelial cells 16HBE and lung cancer cell lines H1299, A549, SPCA1, CALU3 was detected by qRT-PCR. **G**, qRT-PCR analysis of circPTP4A2 expression levels in both H1299 and SPCA1 cells treated with RNase R (**p < 0.01)

To characterize circPTP4A2 expression profiles in NSCLC, we analyzed the circRNA expression profiles of 3 pairs of non-small cell lung cancer patients and their paracancer tissues in the GEO database (GSE158695). The results showed that 84 circRNAs were up-regulated and 100 circRNAs were down-regulated in cancer tissues. The heatmap recapping the expression levels of upregulated and downregulated circRNAs showed that circPTP4A2 was one of the most significantly upregulated ones (|logFC|≥1, *p* < 0.05) (Fig. 1A-B). As shown in Fig. 1C, circPTP4A2 is formed by reverse splicing of exons 1–2 of host gene PTP4A2. To further confirm the upregulation of circPTP4A2, we assessed circPTP4A2 expression in the 50 pairs of NSCLC tissue samples using qRT-PCR and found that circPTP4A2 expression was significantly higher in NSCLC tissues than in the para-cancer tissues (Fig. 1D, *p* < 0.01). Furthermore, we divided the 50 NSCLC patients into high-expression and low-expression groups based on the median circPTP4A2 expression value and conducted a survival curve analysis using KM-plotter. In general, the patients in the high circPTP4A2 expression group had lower survival rates and poorer prognoses than that in the low expression group and the difference was statistically significant (Fig. 1E, log-rank *p* = 0.0499). Patients with high circPTP4A2 expression showed advanced TNM stage, larger tumor size, indicating a more malignant phenotype and worse prognosis (Table S2). Kaplan–Meier survival analysis showed that patients with high circPTP4A2 expression had significantly worse overall survival than those with low circPTP4A2 expression in the public dataset(Figure S3).To examine the effect of circPTP4A2 upregulation on NSCLC progression, we conducted a series of in vitro analyses with four lung cancer cell lines (CALU3, SPCA1, A549, and H1299) and one normal human bronchial epithelial cell line (16HBE). As illustrated in Fig. 1F, qRT-PCR result showed that circPTP4A2 was more highly expressed in several NSCLC cell lines, such as CALU3, SPCA1, A549, H1299 cells, than in the normal human bronchial epithelial cells 16HBE (*p* < 0.01). To provide further evidence to support the structural conformation of circPTP4A2, we treated the extracted total RNA (from H1299 and SPCA1 cell lines) with RNase R and compared the expression of circPTP4A2 in treated and mock cells. The expression level of circPTP4A2 in treated and mock cells was not significantly different, whereas the expression level of linear PTP4A2 was significantly different (Fig. 1G, *p* < 0.01). These findings suggest that circPTP4A2 is upregulated in NSCLC tumors, and a higher circPTP4A2 expression correlates with a worse prognosis.

### Silence circPTP4A2 decreased NSCLC cells proliferation and metastasis in vivo and vitro

**Fig. 2:**
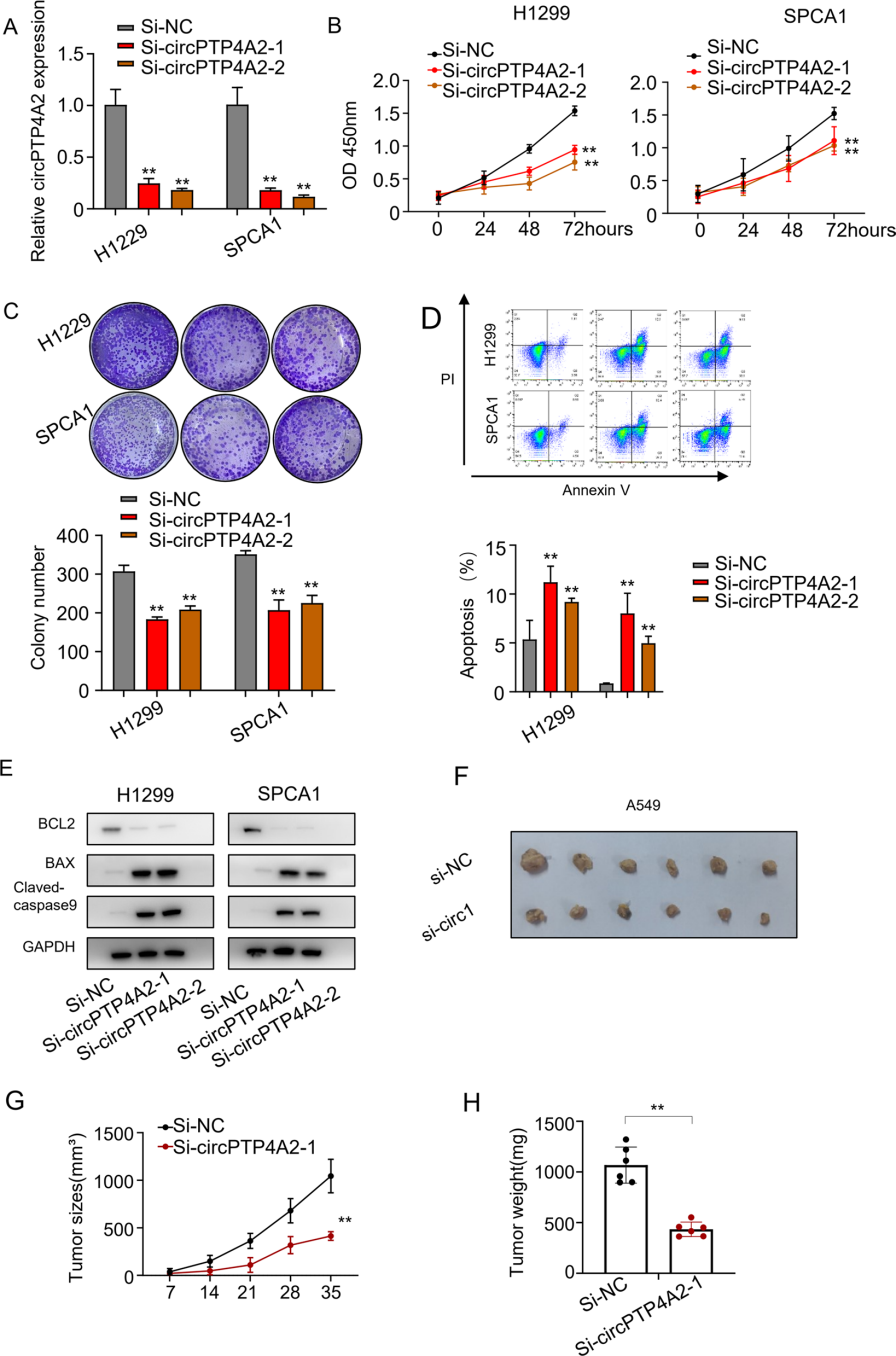
Silence circPTP4A2 decreased NSCLC cells proliferation and metastasis in vivo and vitro. **A**, quantitative real-time polymerase chain reaction confirming the knockdown efficiency of siRNAs in reducing the expression of circPTP4A2 in both SPCA1 and H1299 cell lines. **B**, CCK-8 assay determining cell viability after circPTP4A2 knockdown. **C**, Colony formation assay examining the proliferative ability of the NSCLC cell lines after circPTP4A2 knockdown. **D**, Flow cytometry revealing a significant reduction in the migration of NSCLC cells after circPTP4A2 knockdown. **E**, Western blot analysis of apoptosis markers after circPTP4A2 knockdown. **F-H**, In vivo assay showing the reduction in tumor size, volume, and weight in the mouse models. These parameters were examined at 35 days after injection with sh-circPTP4A2-transfected H1299 cell line (*n* = 6 per group). All experiments were performed in triplicate, and *p* < 0.05 was considered as significant (** *p* < 0.01)

To address how circPTP4A2 expression affects the progression of NSCLC, we conducted a loss-of-function experiment by designing siRNA(small interfering RNA) specifically targeting circPTP4A2 and negative control (Si-NC). Figure 2A reports the knockdown efficiency of the siRNA in reducing the expression of circPTP4A2 in both SPCA1 and H1299 cell lines (*p* < 0.01). The CCK-8 and colony formation assays showed that circPTP4A2 knockdown inhibited the proliferative ability of the NSCLC cell lines and decreased the number of cell colonies formed after transfection compared with the negative control group (Fig. 2B, C, *p* < 0.01).Since the primary cause of death in cancer is often metastasis and deregulation of cell migration during cancer progression can determine the potential of cancer cells to invade para-cancer tissues and form metastases, we assessed the effect of circPTP4A2 on NSCLC cell migration and invasion. In mechanism, we found that circPTP4A2 knockdown could effectively improve the apoptosis ratio of SPCA1 and H1299 cells when the cell was measured by flow cytometry (Fig. 2D). Consistently, western blotting(WB) results showed that knockdown of circPTP4A2 significantly increased the expression of cleaved-caspase 3 and Bax (pro-apoptotic proteins) and decreased the expression of Bcl-2 (anti-apoptotic protein) in A549 and H1299 cells, suggesting that circPTP4A2 knockdown may promote NSCLC cell apoptosis (Fig. 2E, S1). To further examine the effects of circPTP4A2 on NSCLC in vivo, we established a mouse NSCLC xenograft model. As depicted in Fig. 2F-H, the average tumor volume and weights of the harvested tumors from the circPTP4A2 low expressed group was smaller than that from the NC group, especially on day 35 after subcutaneous injection with si-circPTP4A2-transfected H1299 cell line (*p* < 0.01), indicating that knocking down circPTP4A2 could effectively reduce the tumorigenic ability of H1299 cells in tumor-bearing mice. These results suggest that silence circPTP4A2 could decrease NSCLC cells proliferation and metastasis in vivo and vitro.

### CircPTP4A2 direct bonding miR-127-5p

**Fig. 3:**
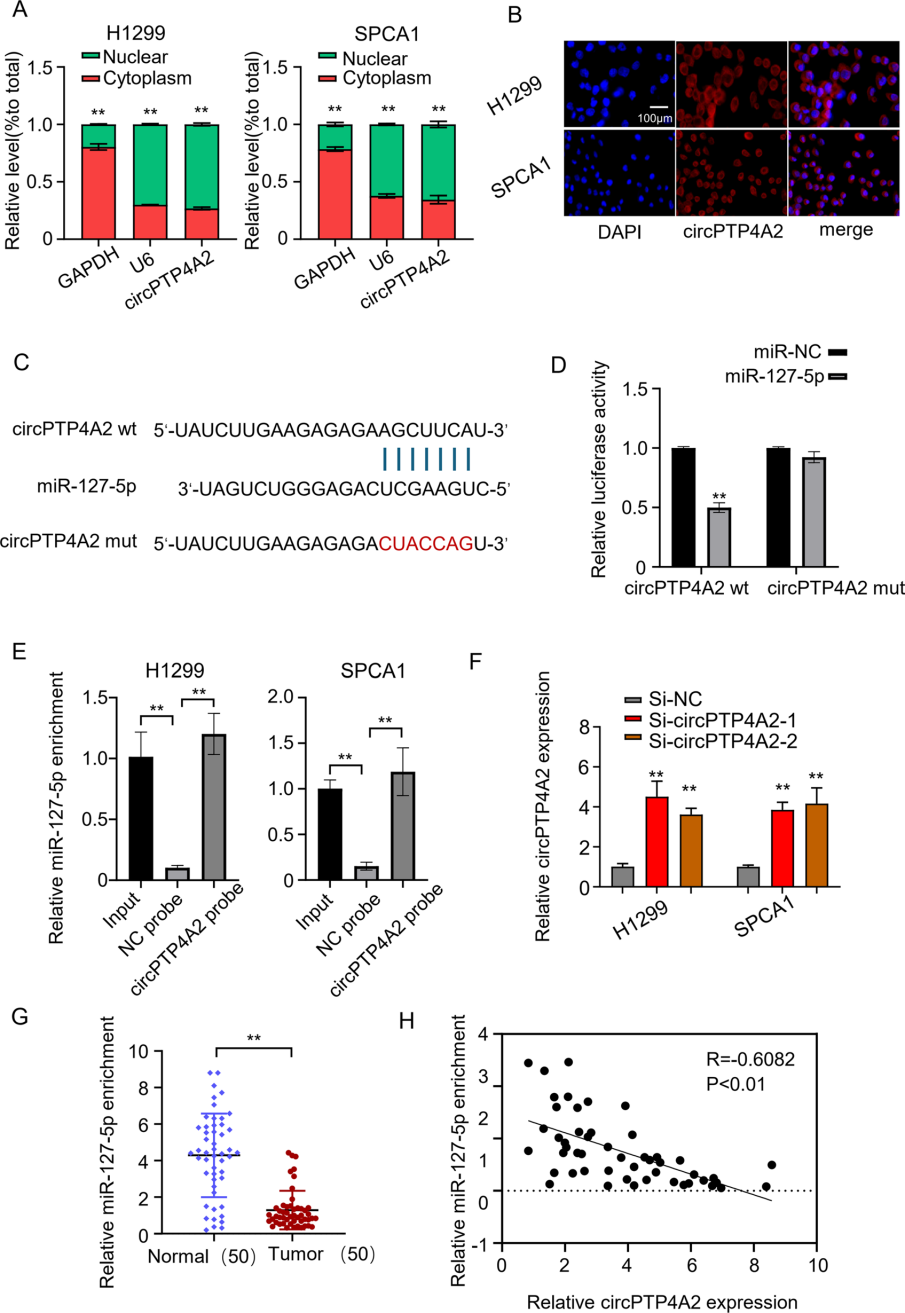
CircPTP4A2 direct bonding miR-127-5p. **A-B**, Nuclear and cytoplasmic separation and fish experiments showed that circPTP4A2 was mainly located in the cytoplasm. **C**, the complementary binding sites of miR-127-5p and circPTP4A2 wild-type (WT) predicted by the online bioinformatics circinteractome. **D**, Dual-luciferase reporter assay to confirm the circinteractome prediction result. **E**, Biotinylated RNA pull-down experiment shows that miR-127-5p was significantly enriched in the circPTP4A2 probe fraction compared with the control probe fraction. **F**, Quantitative real-time polymerase chain reaction (qRT-PCR) analysis reveals that circPTP4A2 knockdown significantly increased miR-127-5p relative expression in the H1299 and SPCA1 cells. **G**, qRT-PCR analysis shows that miR-127-5p is significantly downregulated in NSCLC tissue compared with the control. **H**, Correlation analysis of the expression levels of miR-127-5p and circPTP4A2 in the 50 NSCLC tissues. (**p < 0.01)

For us, it was crucial to decipher that circPTP4A2 can direct bond miR-127-5P. Firstly, we investigated the distribution of circPTP4A2 in vivo, the nuclear RNA and cytoplasmic RNA of SPCA1 and H1299 cells were extracted by the nucleoplasmic RNA extraction kit, and the proportion of circPTP4A2 in each component was detected by qRT-PCR. The results showed that circPTP4A2 was mainly located in the cytoplasm (Fig. 3A). Consistently, the FISH experiment in Fig. 3B also more intuitively demonstrated the localization of circPTP4A2 in the cytoplasm. By using circinteractome, we predicted that circPTP4A2 had a binding site directly binding to miR-127-5P, showed the sequence of binding sites between circPTP4A2 and miR-127-5P, and constructed fluorescein reporter genes from this sequence and the mutant sequence (Fig. 3C). To confirm this prediction, we implemented the luciferase reporter assay using a fluorescent reporter gene containing the WT or the MUT sequence of circPTP4A2.The constructed wild type and mutant fluorescent reporter genes were transfected into 293T cells, and mir-NC and miR-127-5P were transfected, respectively. In the wild-type reporter genes, compared with miR-NC, overexpression of miR-127-5P could significantly inhibit the expression of fluorescein, while in the mutant fluorescent reporter genes, the expression of fluorescein was not affected (Fig. 3D, *p* < 0.01). Next, we conducted the biotinylated RNA pull-down assay to confirm the interaction between circPTP4A2 and miR-127-5P. We constructed circPTP4A2 sequence probe and performed RNA pulldown experiments in SPCA1 and H1299 cells, and the results showed that circPTP4A2 probe could effectively enrich miR-127-5P (Fig. 3E). The expression of miR-127-5P in SPCA1 and H1299 cells after circPTP4A2 knockdown was detected by qRT-PCR. As shown in Fig. 3F that circPTP4A2 knockdown could significantly improve the expression of miR-127-5P. What’s more, we also detected the expression of miR-127-5P in cancer and paracancer samples of 50 pairs of patients with non-small cell lung cancer by qRT-PCR. The results showed that the expression of miR-127-5P in cancer tissues was significantly reduced (Fig. 3G). The expression relationship between circPTP4A2 and miR-127-5P in 50 NSCLC cases was analyzed by person correlation coefficient, and the results showed that the expression of circPTP4A2 and miR-127-5P showed an obvious negative correlation trend (Fig. 3H, *p* < 0.01). These findings suggest that circPTP4A2 can direct bond miR-127-5P.

### miR-127-5P target to SMC3 3’utr and inhibits its expression

**Fig. 4:**
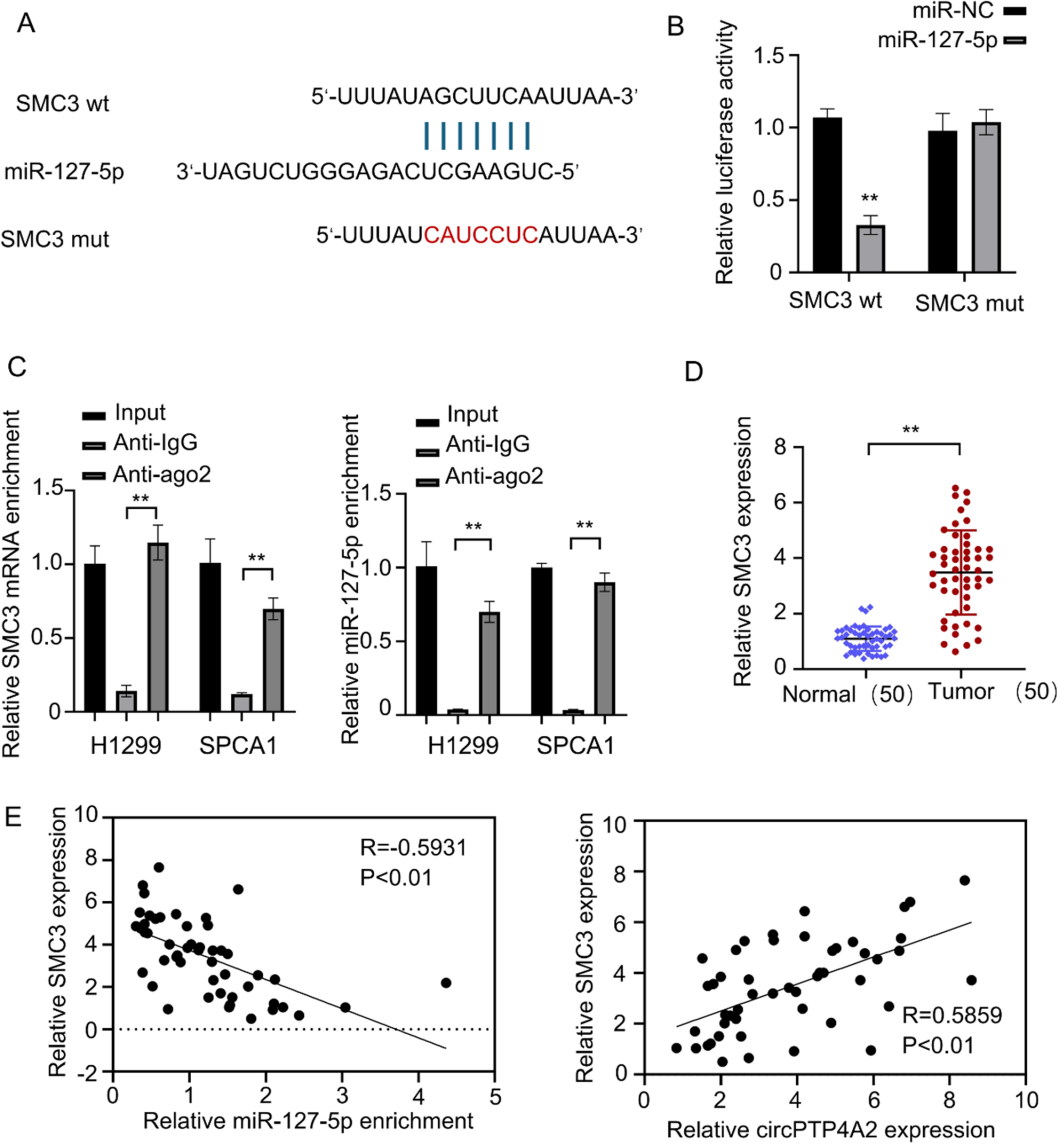
miR-127-5P target to SMC3 3’utr and inhibits its expression. **A**, Schematic representation of the predicted binding site for miR-127-5P in SMC3. The prediction was performed by miRBD. **B**, Dual-luciferase reporter assay confirms the prediction result. **C**, RIP- quantitative real-time polymerase chain reaction (qRT-PCR) analysis shows the enrichment of SMC3 mRNA and miR-127-5P in immunoprecipitated Ago2 relative to IgG. **D**, qRT-PCR analysis of the 50 NSCLC tissues shows that SMC3 mRNA was upregulated in NSCLC tissues compared with the control (Paracancer). **E**, Correlation analysis of the expression levels of SMC3, circPTP4A2, and miR-127-5P in NSCLC tissues. (**p < 0.01)

The function of miRNAs is bimodal, either facilitating or preventing disease progression by regulating target mRNAs. To find the target mRNA for miR-127-5P, we first predicted the candidates using the miRBD (http://mirdb.org/). The prediction revealed that miR-127-5P could bind to the 3 UTR of SMC3. Figure 4 A shows the predicted binding sites of miR-127-5P, in the SMC3 3 UTR. To further substantiate this assertion, 293T cells were transfected with miR-NC and miR-127-5P by constructing wild type and SMC3 sequences mutated and mutated respectively at two binding sites. In wild-type reporter genes, compared with miR-NC, overexpression of miR-127-5P could significantly inhibit the expression of fluoromycin, while in mutant reporter genes, the expression of fluoromycin was not affected (Fig. 4B). What’s more, RIP-qRT-PCR analysis revealed significantly enriched SMC3 mRNA and miR-127-5P in immunoprecipitated Ago2 relative to that in IgG, indicating that both RNAs could interact through the Ago2 complex (Fig. 4C, *p* < 0.01). The expression of SMC3 in cancer and paracancer samples of 50 pairs of NSCLC patients was detected by qRT-PCR. Further analysis revealed that SMC3 was upregulated in NSCLC tissues compared with the control (paracancer) tissues (Fig. 4D, *P* < 0.01), and its expression was positively correlated with the expression level of circPTP4A2 in the NSCLC tissues (Fig. 4D, *p* < 0.01). The expression level of miR-127-5P in NSCLC was negatively correlated with that of SMC3 (Fig. 4E, *p* < 0.01). These findings suggest that miR-127-5P target to SMC3 3’utr and inhibits its expression.

### circPTP4A2 promote NSCLC cells proliferation and metastasis via miR-127-5P/SMC3 axi

**Fig. 5:**
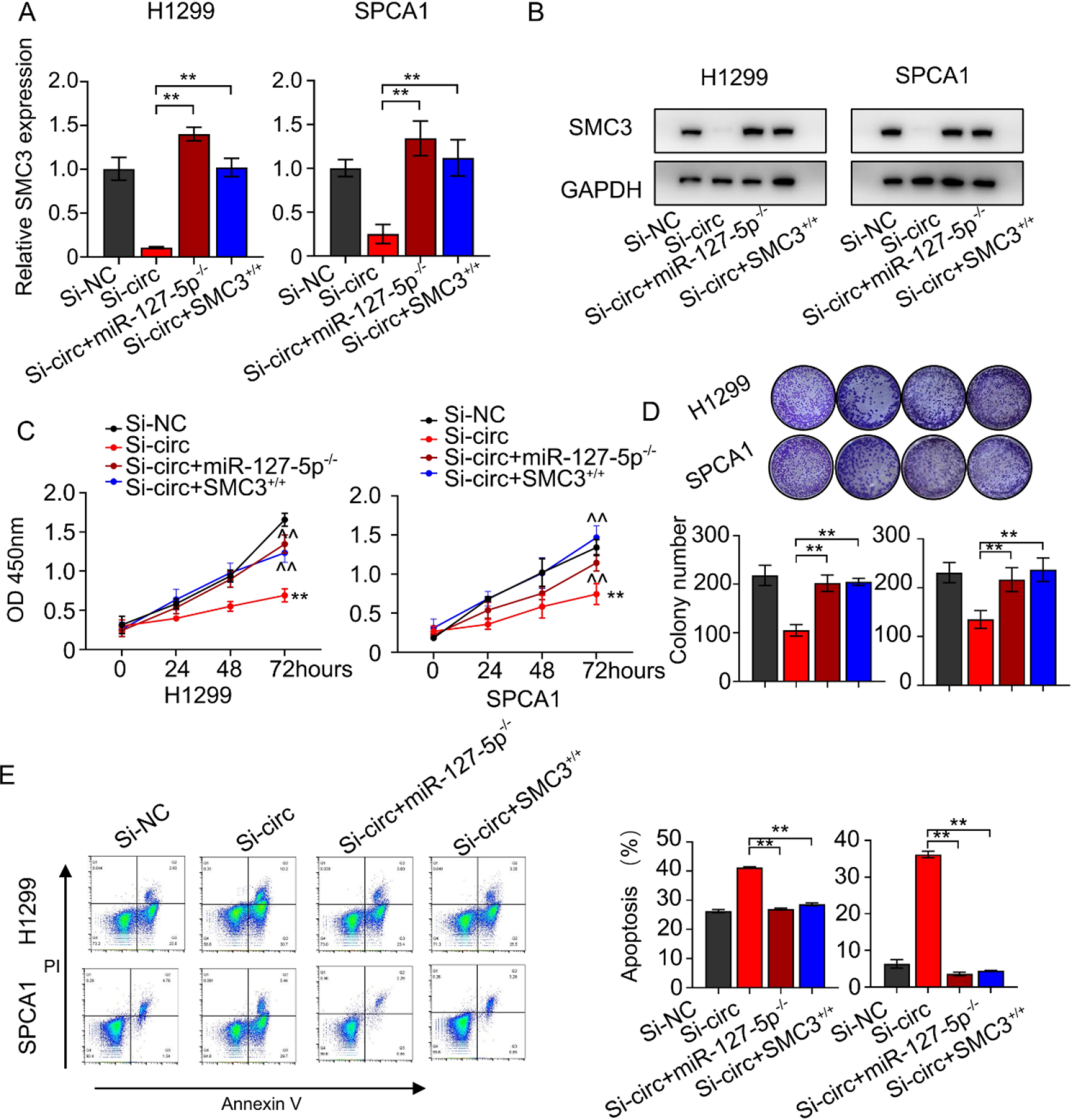
circPTP4A2 promote NSCLC cells proliferation and metastasis via miR-127-5P/SMC3 axi. **A**, quantitative real-time polymerase chain reaction analysis of SMC3 mRNA levels in SPCA1 and H1299 cell lines after circPTP4A2-knockdown, miR-127-5P inhibition, and SMC3 overexpression. **B**, Western blot analysis of SMC3 markers after circPTP4A2-knockdown, miR-127-5P inhibition or SMC3 overexpression. **C**, CCK-8 assay showing that the impaired viability of the circPTP4A2-knockdown cell lines was partially restored by either miR-127-5P inhibition or SMC3 overexpression. **D**, Colony formation assay examining the proliferative ability of the NSCLC cell lines after circPTP4A2-knockdown, miR-127-5P inhibition, and SMC3 overexpression. **E**, Flow cytometry either miR-127-5P inhibition or SMC3 overexpression could reverse the increase of apoptosis ratio caused by circPTP4A2 knockdown (**p < 0.01)

Having demonstrated the association between circPTP4A2, miR-127-5P, and SMC3, we set out to confirm if circPTP4A2 contributes to the development and progression of NSCLC by regulating the miR-127-5P/SMC3 signaling axis. First, we conducted rescue experiments by transfecting the SPCA1 and H1299 cell lines with si- circPTP4A2, miR-127-5P inhibitor, or SMC3 overexpression vector. qRT-PCR showed that knocking down of circPTP4A2 by si-circPTP4A2 was significantly downregulating SMC3 mRNA expression, which could be rescued by either inhibiting miR-127-5P expression or overexpressing SMC3 (Fig. 5A, *p* < 0.01). Consistently, the expression of SMC3 in SPCA1 and H1299 cells after circPTP4A2 knockdown and co-transfection with miR-127-5P inhibitor or overexpression of SMC3 was detected by western blot. As depicted in Fig. 5B, S2 that SMC3 expression decreased after circPTP4A2 knockdown and the expression of SMC3 was recovered after co-transfection with miR-127-5P inhibitor or SMC3+. CCK8 detected the cell activity changes of SPCA1 and H1299 cells after circPTP4A2 knockdown and co-transfection of miR-127-5P inhibitor or overexpression of SMC3.Notably, co-transfection with miR-127-5p inhibitor or SMC3 + can effectively reverse the cell activity reduction induced by circPTP4A2 knockdown (Fig. 5C). In addition, we examined the changes in the clonogenesis capacity of SPCA1 and H1299 cells after circPTP4A2 knockdown and co-transfection with miR-127-5P inhibitor or overexpression of SMC3. The results showed that co-transfection with miR-127-5p inhibitor or SMC3 + can effectively reverse the reduction of clone formation ability induced by circPTP4A2 knockdown (Fig. 5D). In addition, flow cytometry was used to detect the apoptosis ratio of SPCA1 and H1299 cells after circPTP4A2 knockout and co-transfection of miR-127-5P inhibitor or overexpression of SMC3. As depicted in Fig. 5E, co-transfection with miR-127-5p inhibitor or SMC3 + can effectively reverse the increase in apoptosis ratio induced by circPTP4A2 knockdown. Collectively, these findings suggest that circPTP4A2 promote NSCLC cells proliferation and metastasis via miR-127-5P/SMC3 axi.

## Discussion

Non-small cell lung cancer (NSCLC) remains one of the most common and lethal malignancies worldwide. Accumulating evidence has linked aberrant circRNA expression to tumorigenesis, including NSCLC. However, the functional roles of circRNAs in NSCLC pathogenesis remain largely unclear. In this study, we identified and characterized a novel oncogenic circRNA, circPTP4A2 (hsa_circ_0007364), which is generated by circularization of exons 1–2 of the PTP4A2 gene. PTP4A2 has previously been implicated in tumor progression and stem cell self-renewal. We found that circPTP4A2 was significantly upregulated in NSCLC tissues and cell lines, and its high expression predicted poor patient survival. Functional experiments revealed that circPTP4A2 promoted NSCLC cell proliferation, migration, invasion, and inhibited apoptosis in vitro, as well as tumor growth in vivo.

Mechanistically, circRNAs frequently function as miRNA sponges in cancer. Bioinformatic prediction and experimental validation confirmed that circPTP4A2 directly binds and sequesters miR-127-5p, a well-documented tumor suppressor in multiple cancer types. In line with previous studies, miR-127-5p was downregulated in NSCLC tissues and inversely correlated with circPTP4A2 expression. Inhibition of miR-127-5p rescued the anti-tumor effects of circPTP4A2 knockdown, confirming the sponge role of circPTP4A2.

We further identified SMC3 as a direct downstream target of miR-127-5p. SMC3 is a core component of the cohesin complex and is dysregulated in various cancers. SMC3 was upregulated in NSCLC and positively correlated with circPTP4A2 expression, while negatively correlated with miR-127-5p. Overexpression of SMC3 reversed the inhibitory effects of circPTP4A2 silencing on NSCLC progression. These findings establish the circPTP4A2/miR-127-5p/SMC3 regulatory axis in NSCLC.

Notably, circPTP4A2 has been reported to promote cervical cancer via the miR-101-5p/MAT2A axis; our study is the first to reveal its oncogenic role and specific mechanism in NSCLC. The newly identified axis connects circPTP4A2 to SMC3, a key regulator of chromosomal stability and tumor progression, expanding the landscape of circRNA-mediated regulatory networks in NSCLC. Clinically, circPTP4A2 may serve as a promising prognostic biomarker and therapeutic target for NSCLC. This study has some limitations. The sample size (*n* = 50) is relatively small, and the survival result was marginally significant. However, analysis of a public dataset supported the prognostic value of circPTP4A2. Further validation in large, multi-center cohorts is warranted to confirm its clinical application.

## Supplementary Information

Below is the link to the electronic supplementary material.

Supplementary Material 1

## Data Availability

No datasets were generated or analysed during the current study.
