# Supplementary material for: CircPTP4A2 (hsa_circ_0007364) facilitates non-small cell lung cancer progression by regulating miR-127-5p/SMC3
Source: Biol Direct. 2026 May 7;21:115. doi: 10.1186/s13062-026-00815-2 (PMC13321512; doi:10.1186/s13062-026-00815-2)
Supplement: Supplementary file 1 — Supplementary Material 1 [file 13062_2026_815_MOESM1_ESM.docx]

**Supplementary materia**


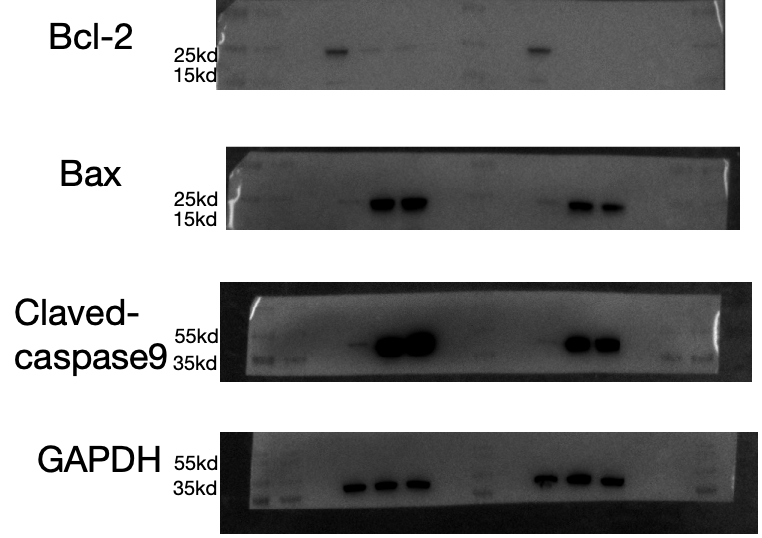


**Figure S1:** Complete uncropped gel and blot images.


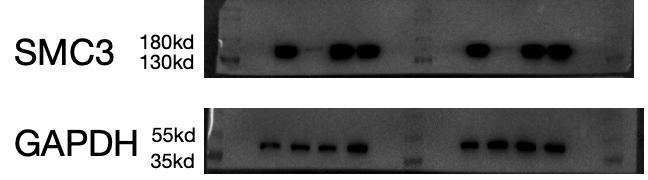


**Figure S2:** Complete uncropped gel and blot images.


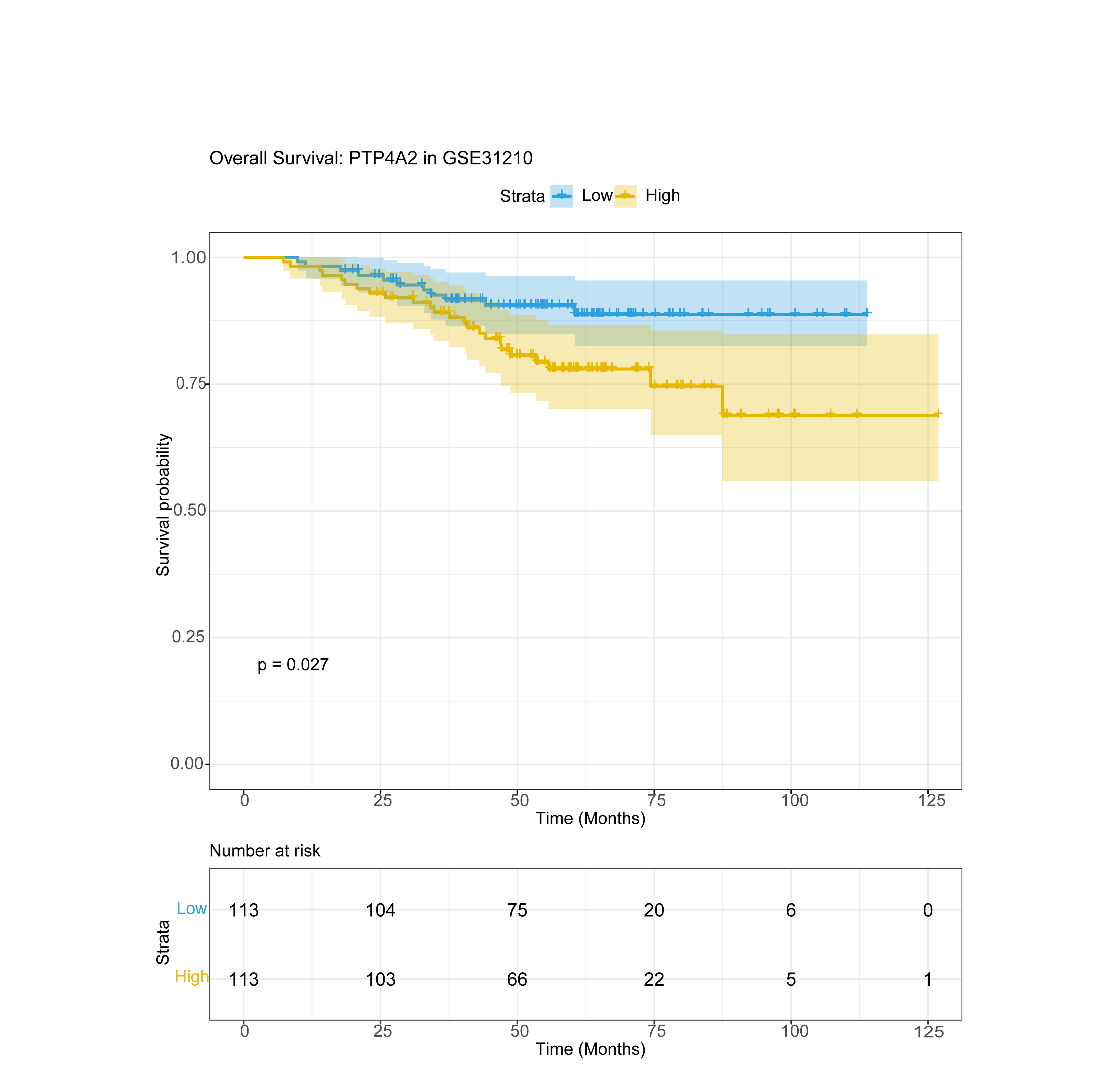


**Figure S3:** Public database GSE31210.

Table S1

| **Primers** | **Sequence** |
| --- | --- |
| Primers for circPTP4A2 | F: 5’-GGAGTGACGACTTTGGTTCG-3’ |
|  | R:5’-TGTCAGCGAAAATGCTGTGC-3’ |
| SiRNA-1 for circPTP4A2 | 5’-[AGGAATCCACGTTCTAGTTTT](http://blast.ncbi.nlm.nih.gov/Blast.cgi?PROGRAM=blastn&PAGE_TYPE=BlastSearch&LINK_LOC=blasthome&QUERY=%3Ehsa_circ_0007364-siRNA1%0AAGGAATCCACGTTCTAGTTTT&DATABASE=nr&EQ_MENU=Homo%C2%A0sapiens%C2%A0(taxid:9606))-3’ |
| SiRNA-2 for circPTP4A2 | 5’-ATCCACGTTCTAGTTTTTCGT-3’ |
| Primers for miR-127-5P | F:5’-GGAAGATCTGTAGTCCTGTCTGTTGGTCAG-3’ |
|  | R:5’-CCCAAGCTTCCTGAAGAACTGCTTCCGCC-3’ |
| Primers for SMC3 | F:5’- ATCTTCGTCCAGAACAGCGG-3’ |
|  | R:5’- GTGCTGTTGCCATCTGGTTG-3’ |

Table S2 -Correlation between circPTP4A2 expression and clinicopathologic features of 50 patients.

| **Characteristic** | **Total (n=50)** | **circPTP4A2 High Expression (n=25)** | **circPTP4A2 Low Expression (n=25)** | **P-value** |
| --- | --- | --- | --- | --- |
| Age,years | 61.5 ± 8.8 (mean ± SD) | 63.1 ± 8.2 (mean ± SD) | 59.9 ± 9.3 (mean ± SD) |  |
| Gender, n (%) |  |  |  |  |
| Male | 32 (64.0) | 18 (72.0) | 14 (56.0) |  |
| \| Female \| \| --- \| | 18 (36.0) | 7 (28.0) | 11 (44.0) |  |
| Smoking history, n (%) |  |  |  |  |
| Never smoker | 21 (42.0) | 8 (32.0) | 13 (52.0) |  |
| Former/current smoker | 29 (58.0) | 17 (68.0) | 12 (48.0) |  |
| Pathological type, n (%) |  |  |  | 0.018 |
| Adenocarcinoma | 33 (66.0) | 22 (88.0) | 11 (44.0) |  |
| Squamous cell carcinoma | 17 (34.0) | 3 (12.0) | 14 (56.0) |  |
| Tumor size, cm | 4.5 ± 1.6 (mean ± SD) | 5.3 ± 1.4 (mean ± SD) | 3.7 ± 1.5 (mean ± SD) | < 0.05 |
| TNM stage, n (%)（TNM分期） |  |  |  | < 0.05 |
| Ⅰ/Ⅱ | 15 (30.0) | 2 (8.0) | 13 (52.0) |  |
| Ⅲ/Ⅳ | 35 (70.0) | 23 (92.0) | 12 (48.0) |  |
| Differentiation, n (%) |  |  |  | < 0.05 |
| Well/Moderately differentiated | 18 (36.0) | 3 (12.0) | 15 (60.0) |  |
| Poorly differentiated | 32 (64.0) | 22 (88.0) | 10 (40.0) |  |
| Lymph node metastasis, n (%) |  |  |  | < 0.05 |
| Yes | 33 (66.0) | 24 (96.0) | 9 (36.0) |  |
| No | 17 (34.0) | 1 (4.0) | 16 (64.0) |  |
